# Swellable catheters based on a dynamic expanding inner diameter

**DOI:** 10.1007/s10856-021-06524-8

**Published:** 2021-04-23

**Authors:** Rishabh Tennankore, Margaret Brunette, Tyler Cox, Rigoberto Vazquez, Ariella Shikanov, Michael L. Burns, Brian Love

**Affiliations:** 1 Department of Material Science & Engineering, University of Michigan, Ann Arbor, USA; 2 Department of Biomedical Engineering, University of Michigan, Ann Arbor, USA; 3 Department of Aerospace Engineering, Iowa State University, Ames, USA; 4 Department of Nuclear Engineering & Radiological Science, University of Michigan, Ann Arbor, USA; 5 Department of Anesthesiology, University of Michigan, Ann Arbor, USA

## Abstract

Intravenous (IV) fluid administration is critical for all patients undergoing care in a hospital setting. In-patient hospital practice, surgeries, and emergency care require functional IVs for fluid replacement and medication administration. Proper placement of IVs is vital to providing medical services. The ease of placement of an IV catheter, however, depends not only on the size of the catheter but also on provider experience and patient demographics such as age, body mass index, hydration status, and medical comorbidities present challenges to successful IV placement. Smaller diameter IV placement can improve success and there are instances where multiple small diameter catheters are placed for patient care when larger bore access is unattainable. Smaller inner-diameter catheters for anesthesia have functional constraints. Ideally, there would be a smaller catheter for placement that could function as a larger catheter for patient care. One solution is the idea of functionally responsive catheters. Here, we evaluated tubular-shaped hydrogels as potential functional catheters that can increase in inner diameter through fluid swelling using cross-linked homopolymers of polyacrylamide, PAM (10–40% w/w), and their copolymers with 0–8% w/w Poly-(Ethylene Glycol)-Diacrylate, PEGDA. For the PAM gels, the water transport mechanism was shown to be concentration-dependent Fickian diffusion, with the less concentrated gels exhibiting increasingly anomalous modes. Increasing the PEGDA content in the network yielded an initial high rate of water uptake, characterized by Case II transport. The swelling kinetics depended strongly on the sample geometry and boundary conditions. Initially, in a submerged swelling, the annulus expands symmetrically in both outward and inward directions (it thickens), reducing the internal diameter by up to 70%. After 1 h, however, the inner diameter increases steadily so that at equilibrium, there is a net (>100%) increase in all the dimensions of the tube. The *amount* of linear swelling at equilibrium depended only on the polymer volume fraction as made, while the *rate* of inner diameter expansion depended on the hydrophilicity of the matrix and the kinetics of sorption. This study serves as proof of concept to identify key parameters for the successful design of hydrogel-based catheter devices with expanding inner-diameters for applications in medical care.

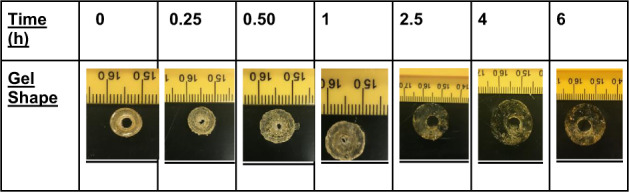

## Introduction

Every year over 200 million peripheral IV catheters are placed in the USA [1]. These placements come at a cost. A 2013 study found the median cost of a pediatric IV insertion to be valued at $5.50 per supply set used in each IV placement [2] and 16–33% of IV attempts fail [3–5]. While there are many reasons for potential failed IV placement (such as obesity, edema, dehydration, positioning, discomfort, patient comorbidities, etc.), the size of the IV cannula being placed is also relevant. Larger IVs allow for higher throughput but can be difficult to place and cause more pain to the patient during placement [5]. Smaller diameter IVs are easier to place, and often when a difficult placement is anticipated, a smaller IV may be placed with the hopes of placing a second larger IV after initial successful placement. This lower IV access may be ineffective for patient care. The goal of this study is to probe functionally responsive hydrogel-based catheters capable of swelling to yield a larger internal diameter (ID) after placement into an aqueous environment (such as a patient’s vein). This larger ID could allow higher flow rates for administering medications, fluids, and/or blood products, as is often needed clinically.

There are no functionally responsive catheters available, but, there have been attempts to create similar clinical devices in the past. Most notably, Menlo Care created “enlarging IV catheters” marketed as Streamline® catheters, around 1989. These catheters were formed by co-extruding a multilayer cannula structure comprising an outer layer made of a hydrophilic component such as polyvinyl alcohol, poly-(ethylene oxide), poly-(ethylene glycol), etc., and a relatively hydrophobic component, commonly polyurethane [6]. Results published in the literature showed a lower expansion of a possible propensity to kink when placed in vivo [7–9]. However, these catheters were well received after commercialization. Becton-Dickinson and Company also reported a functional catheter, designed using only a *single* hydrophilic, elastomeric copolymer containing ‘hard’ and ‘soft’ segments to confer both mechanical durability and hydrophilicity to the part. The ‘hard’ non-hydrophilic and ‘soft’ hydrophilic segments used were reported to be an isocyanate such as methylene diisocyanate and a high molecular weight polyethylene glycol (PEG) [10]. Previous attempts were hindered by either mechanical durability issues and/or biocompatibility issues. Most concerning, the deployment of Landmark midline catheters made of Aquavene® (copolymer of PU and PEO) were alleged to have caused life-threatening adverse hypersensitivity reactions [11]. These allegations were controversial, and while the exact cause of the reactions was never discovered, products containing Aquavene® were subsequently pulled from the market. To circumvent the issues from previous attempts, we chose to evaluate networks of polyacrylamide (PAM) and hydrophilic poly(ethylene glycol) diacrylate (PEGDA) as swellable matrices because of their widely reported biocompatibility [12–14] and remarkable mechanical toughness [15]. We wanted to assess the kinetic expansion of these polymers and compare it with the materials composing the midline catheters deployed years ago.

To successfully function as a catheter, a hydrogel needs to swell and transform rapidly in a hydrophilic environment (hours vs days). Therefore, it is essential to characterize the water–polymer interactions and the dynamics of swelling in any candidate material. Based on the relative rates of penetrant diffusion and polymer chain relaxation, there are three models that describe the swelling responses of hydrophilic polymer networks [16], regulated by a time of sorption, relative to a characteristic time regulated by the polymer.

Case I—Fickian Diffusion occurs when *t*_d_ ≫ *t*_r_

Case II—when *t*_r_ > t_d_, case II diffusion occurs

Case IIl—When *t*_r_ ~ *t*_d_, anomalous diffusion occurs i.e. the kinetics are defined by some combination of diffusion and relaxation processeswhere *t*_r_ is the time taken for polymer chain relaxation, and *t*_d_ is the time taken for the diffusion of solvent penetrant. The diffusion mechanism can be found by gravimetric water sorption, where the mass of the water-swollen polymer, *M*_*t*_, is tracked over time, *t*. When *M*_*t*_ is normalized to the swollen polymer at its equilibrium hydration level, *M*_∞_, the transport mechanism is shown as

1 $$\frac{{M_t}}{{M_\infty }} = kt^n$$

where *k* and *n* are constant for a particular drug delivery system. However, this semi-empirical relation is valid only for the first 60% of normalized water uptake. For a cylindrical sample geometry, a value of *n* = 0.451 ± 0.004 is indicative of Case I or Fickian diffusion, while a value of *n* = 0.89 ± 0.02 indicates Case II diffusion, defined by an initial linear fractional uptake. Lastly, any value of *n*, between these two extremes indicates anomalous diffusion [17, 18]. The work presented here probes the synthesis, characterization, and water transport through homo- and hetero-networks of PAM and PEGDA.

Our objectives were (1) cast PEGDA materials as tubular gels, (2) quantify swelling behavior of these tubular gels to understand their sorption kinetics and diffusion parameters. In characterizing this material, we aim to identify an alternative to Aquavene^®^ for use in functionally responsive IV catheters. This early work can be instrumental in redesign, serving as a potential solution to difficult IV catheter placement in the medical care setting.

## Materials and methods

### Materials

The monomer acrylamide, AAm, initiator Ammonium Persulphate, APS, and crosslinkers—N,N′-methylene-bis(acrylamide), MBA, and Poly(Ethylene Glycol)-Diacrylate, PEGDA (*M*_n_ = 700 g/mol), were all purchased from Sigma Aldrich. All the chemicals were used as purchased without any further purification.

### Sample preparation

All gels were prepared using deionized (DI) water that was degassed by N_2_ purge. The monomer, AAm, was dissolved in water to a concentration of 10–40% w/w w.r.t final weight of the solution. Keeping the total polymer content in the solution fixed at 20% w/w, the amount of PEGDA was varied between 0 and 8% w/w w.r.t final weight of solution. The solution was then vigorously vortex mixed to ensure complete dissolution. The initiator APS was then added at a concentration of 0.5% w/w w.r.t. the final weight of solution. The crosslinker ratio, *X*, (moles of crosslinker / moles of Acrylamide) was maintained between 0.013 and 0.071 in all the networks. All the acrylamide homopolymers were synthesized using MBA (*X* = 0.013) as a crosslinker, while the heterogeneous networks rely on PEGDA (*X*_min_ = 0.013, *X*_max_ = 0.071) and acrylamide to both advances via alkene radical crosslinking. Once the initiator is solubilized, the solution was transferred into a mold and placed into an oven preheated to 70 °C, for 30 min for a quick thermal cure. Subsequently, the formed gels were removed from the mold and washed with IPA to remove any unreacted monomer. Using an X-acto knife, the gel was cut into desired lengths and dried in a vacuum oven maintained at 80 °C for 24 h. Samples were formed with between 10 and 90% PEGDA, but samples with >10% PEGDA embrittled and formed cracks during dehydration. Samples with more than 20% PAM:PEGDA also cracked during dehydration. Samples less than 15% survived the initial dehydration but cracked during swelling experiments typically before 48 h of immersion.

### Water sorption measurements

For the data shown in Figs. 2 and 3, the tube-like annulus gels (OD = 8 mm, ID = 3 mm) were immersed in beakers containing 40 mL DI water, at *t* = 0. The gels absorbed water and swelled as a function of time. The beakers were periodically replenished with water to maintain a constant water level throughout the swelling experiment. At predetermined time intervals, the polymer tubes were removed from the water bath, any excess water was blotted off using an absorbent wipe, and their masses and dimensions (OD, ID, and H) were recorded. ImageJ software was used to measure gel dimensions at each time interval. The amount of water absorbed (g) and the average dimensions of the gel (mm) were monitored over 48 h.

#### Statistical analysis

Data are presented as mean ± standard error. The standard error was calculated based on three replicates (*n* = 3). The repeated measure analysis of variance (ANOVA) technique was used to compare the gravimetric swelling data sets (*N* = 30) because the measurements corresponded to the same sorption time intervals in each network. Pairwise Student’s *T* tests (two tailed, paired samples) were done subsequently for individual comparisons. On the other hand, for the linear swelling data sets, a two-tailed, paired sample *T*-test was directly used to compare the dimensional changes for the treated networks and untreated (homopolymer) samples. *P* values less than 0.05 were considered significant.

## Results and discussion

### Casting tubular gels

**Fig. 1:**
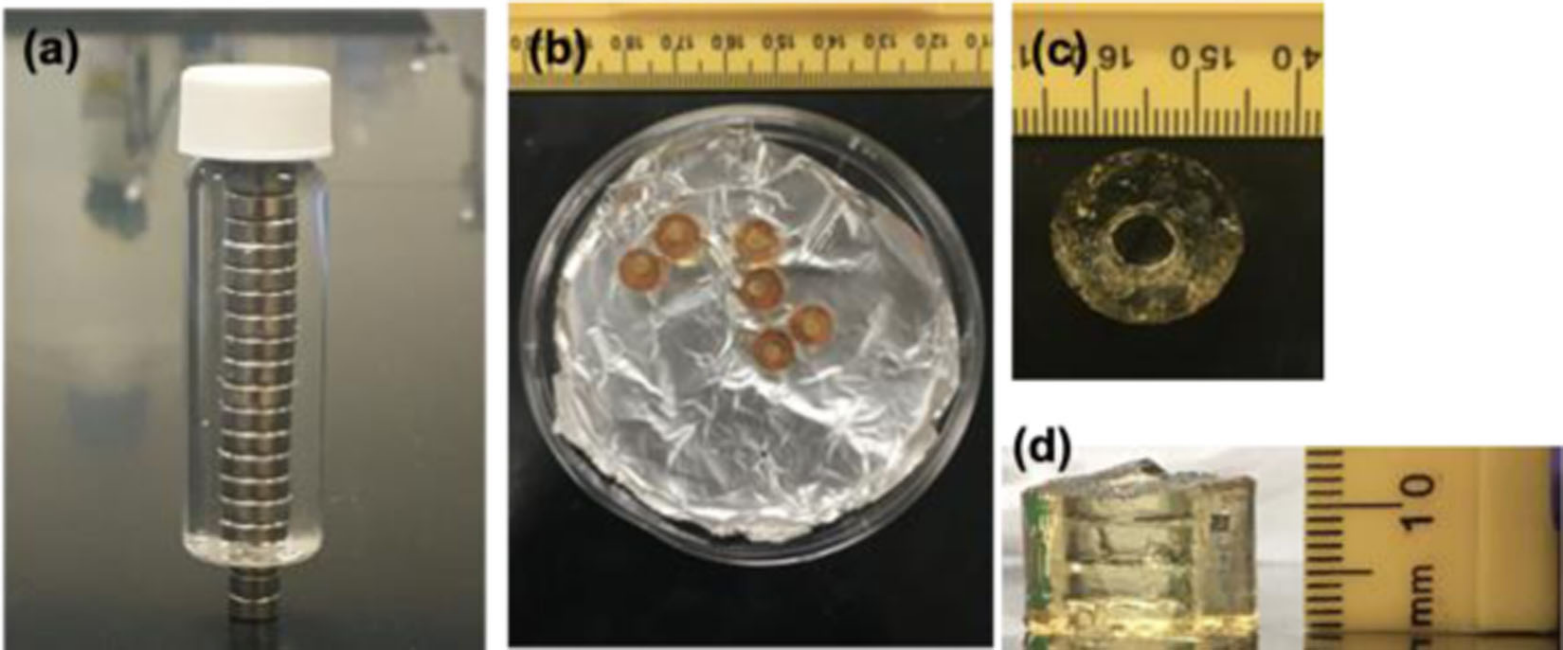
**a** Annular mold—a magnetic bead column positioned centrally inside a standard 10 mL scintillation vial; **b** vacuum dried gels; **c** swollen gel Diameter and **d** height measurement using ImageJ

Our scheme for the preparation and characterization of the tube-like annulus gels is illustrated in Fig. 1. We use a hollow mold, the simplest version is made by arranging a small cylinder such as a vial or tube, concentrically inside a bigger cylinder. Here a standard Fisherbrand^™^ borosilicate 10 mL scintillation vial was used as the big cylinder. A central column of button-sized (*D* = 2.5 mm) neodymium magnets is used to make up the smaller cylinder. The reaction mixture is then pipetted into the annular space between the magnetic column and vial. After a short thermal cure, the glass vial is sacrificed and the magnetic column easily slides out of the formed gel. The formed gels have consistent dimensions as was required for our study because they assume the dimensions of the mold. This type of sacrificial molding technique has been successfully used to create hydrogel-based 3D scaffolds for tissue engineering in the past as well [19].

## Water sorption studies

### Swelling behavior

**Fig. 2:**
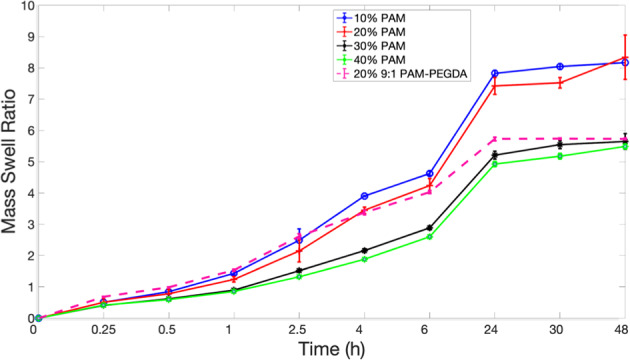
Sorption curves for the diffusion of water at 25 °C into PAM homopolymer networks (solid line) vs PAM-PEGDA network (dashed line) (For the observed values of swelling ratios in the following networks: *P* < 0.0001; calculated using the repeated measure ANOVA)

PAM/PEGDA hydrogels of tubular geometry, with 10–40% w/w monomer concentrations, were fully immersed in water at 25 °C (RT) for 48 h and their water uptake was monitored (Fig. 2). The mass swelling ratio, *Q*_M_, and water diffusivity were calculated from these data. *Q*_M_ is simply defined as the ratio of the mass of water absorbed at time ‘*t*’ to the mass of dry polymer. For PAM homopolymers, the rate of solvent uptake varied inversely with the concentration of monomer. Ten percent PAM & 40% PAM had the highest and lowest swelling ratios (782% and 492%) at equilibrium, respectively. As polymer concentration increases in the network, there are more adjacent acrylamide groups. A more concentrated gel leads to a higher effective crosslink density, which lowers swelling. This relation between monomer % and the swelling ratio has been previously reported for other polymers as well [20]. In contrast, the swelling ratio of the PEGDA hetero-network at equilibrium was lower, between that of 20% PAM and 30% PAM. The lower swelling response at equilibrium is linked with the heterogeneous network formed from chain-growth polymerization. Because the monomers are cross-linked via polymer kinetic chains, CG polymerization offers less control over crosslink functionality, and network defects such as chain entanglements are more likely. It has even been reported in the past that step growth polymerized PEG hydrogels display lower stiffness and greater swelling compared to CG polymerized gels [21]. Despite this, however, the PEGDA network still exhibits the highest mass, and corresponding linear swelling ratios in the first 2.5 h of swelling (Fig. 2). It has also been proven in previous studies that using longer PEGDA chains can not only increase the swelling ratio but also yield a higher tensile modulus. For instance, using a PEG 17-mer as crosslinker over a tetramer, has raised elastic modulus 150% [22]. Thus, one can optimize both mechanical strength and mass swelling ratio in a PEG-based hydrogel by varying the mode of polymerization and tuning the concentration and molecular weight of the PEG crosslinker.

**Fig. 3:**
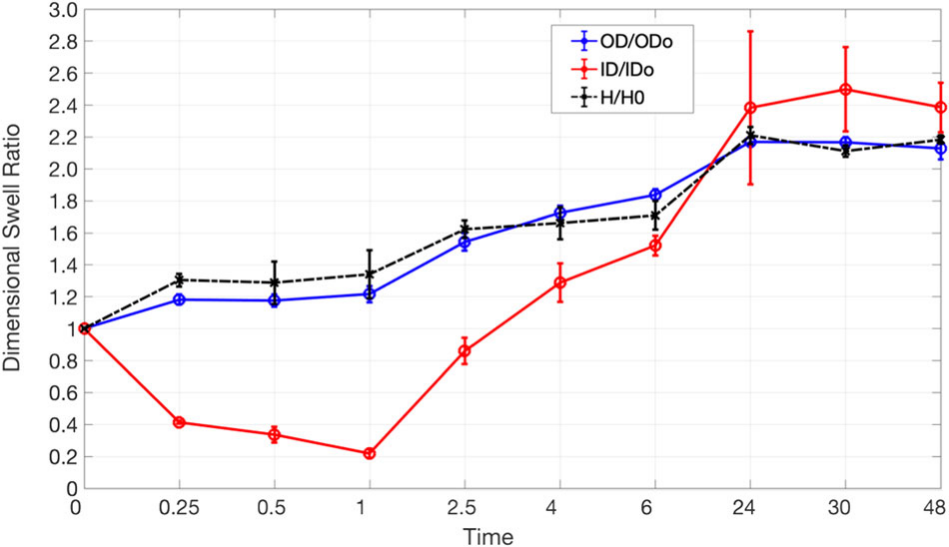
Variation of linear swelling ratios, OD_t_/OD_o_, ID_t_/ID_o_, and *H*_t_/*H*_o_ with Time for a 20% PAM homopolymer network immersed in DI water for 48 h

**Fig. 4:**
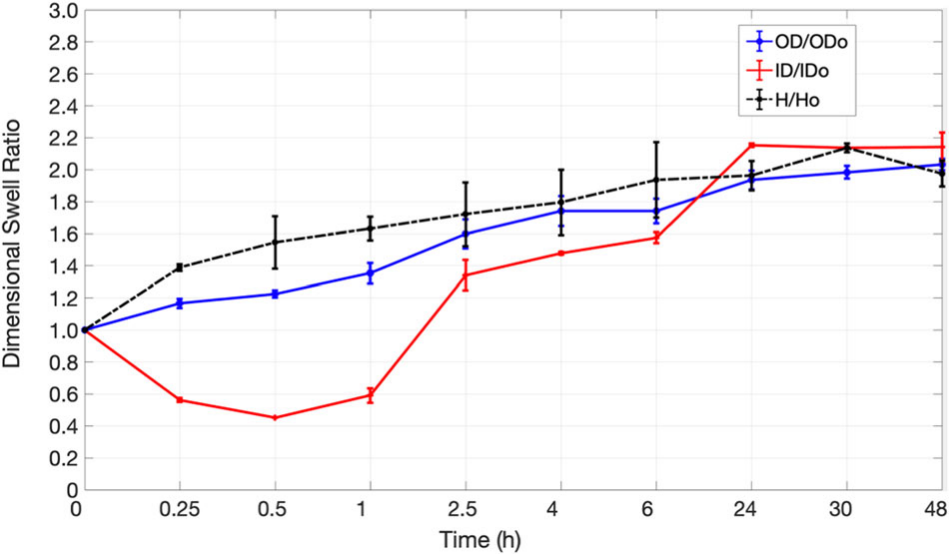
Variation of linear swelling ratios, OD_t_/OD_o_, ID_t_/ID_o_, and *H*_t_/*H*_o_ with Time for a 20% 9:1 PAM-PEGDA network immersed in DI water for 48 h

The homopolymer tubes maintained their cylindrical shape during the 48-h swelling, so one could measure the average gel dimensions at each time point using ImageJ software. In contrast, only those network tubes that contained <4% w/w PEGDA, maintained their structural integrity when hydrated. Above that concentration, cracks formed at the outer boundary, propagating inwards. This is likely because, at higher concentrations of PEGDA, the effective crosslink density of the network increases so much that the differential time rates of expansion in OD and ID results in catastrophic failure. Figures 3 and 4 show the variation of linear swelling ratios over 48 h for 2 polymer networks—20% w/w AAm homopolymer, MBA (*X* = 0.013); 20% w/w AAm-PEGDA network, PEGDA (*X* = 0.013). No significant statistical difference was seen between their rates of linear swelling at equilibrium. This is in accordance with a scaling relationship based on the Flory–Rehner equilibrium swelling theory. It was reported that when the polymer volume fraction in the prepared state, *v*_2_^0^, was greater than 0.1, the linear swelling ratio ‘*ɑ*’, increased with increasing *v*_2_^0^ [23]. To determine *v*_2_^0^, the prepared PAM-PEGDA gels were dried to constant mass in an oven maintained at 80 °C for 24 h, and *v*_2_^0^ was calculated according to the formula:

2 $$v_2^0 = \left[ {1 + \frac{{(q_{\rm{F}} - 1)\rho }}{{d_1}}} \right]^{ - 1}$$

where *q*_**F**_ is the dilution degree after gel preparation (mass of gel as cast/mass of dried gel), *ρ* is the polymer density and *d*_1_ is the solvent density (1 g/mL for water). Table 1 shows the calculated values of *q*_F_ and *v*_2_^0^ for each network. Raising the monomer content in the prepolymer mixture led to a higher *v*_2_^0^.

**Table 1:** Degree of dilution & polymer volume fraction in prepared gels

Polymer	*q*_F_	*v*_2_^0^
PAM 10%	8.109 ± 0.046	0.112 ± 0.00064
PAM 20%	4.156 ± 0.0206	0.222 ± 0.0011
PAM 30%	2.846 ± 0.0312	0.328 ± 0.0037
PAM 40%	2.106 ± 0.0616	0.449 ± 0.0135
PAM-PEGDA 20% (2% PEGDA)	4.182 ± 0.0146	0.22 ± 0.00078

Second, both the 20% PAM homopolymer and 9:1 20% PAM-PEGDA networks had the same polymer volume fraction. This shows that the linear swelling ratio at equilibrium must depend solely on the polymer volume fraction, regardless of the individual polymers’ sorption capabilities. However, within a 6-hour window, the PEGDA network displayed larger ID growth as compared to the homopolymer network (*P* = 0.00038), while there was no significant difference in their rates of OD growth (Figs. 3 and 4). The following subsections address this distinctive behavior. It was also seen that not only the OD and ID, but the lengths of the hydrogels almost doubled after 48 hours as well.

From Figs. 3 and 4, in the time interval 0 < *t* < 1 h, ID shrinks by up to 70% before any kind of growth occurs, while the OD expands monotonically till equilibrium. This shows that the growth in ID lags behind the growth in OD. Increasing the fraction of PEGDA content in the polymer results in greater initial water uptake, faster ID recovery, and reduces lag time. For instance, at 2.5 hours, the PAM homopolymer exhibits an ID that is 25% smaller than the initial dimension. In contrast, even at this short-time scale, the ID of the PEGDA network has actually grown by 34%. Employing PEGDA chains of greater molecular weight as crosslinkers (keeping *X* and *v*_2_^0^ the same) can not only yield higher rates of diffusion and swelling ratios as reported by others in the past but can also reduce the lag time for ID expansion. The initial ID shrinkage is linked with sample shape symmetry and the corresponding boundary conditions in place. Maybe shrinkage can be optimized if either the outer or inner surfaces are made impermeable to solvent penetration for instance. However, it is important to note that doing so would also alter the boundary conditions for normalized water uptake that are used to describe diffusion kinetics.

Comparing the dynamic expansion characteristics with the Aquavene catheters is not exactly clear. Prior work focused on increased flow which was noted within one hour of installation and doubled within 2 h. The experiments here were not under a gravity-driven flow, but the kinetics of expansion are definitely slower than with Aquavene [7]. A legitimate question is what is an optimum swelling ratio? On a dimensional basis, the reality is that profound swelling also results in lower mechanical integrity. If the volumetric flow rate could double, that seems achievable without compromising the integrity of the tube to avoid kinking and sagging. A dimensional change for the inner diameter of 2 seems optimum and could be further refined down if necessary. Our focus here was simply a demonstration of what was potentially feasible.

### Sorption kinetics

The first step in diffusion through a glassy polymer is the dispersal of penetrant molecules across the polymer surface. It is usually assumed that the interface reaches equilibrium concentration instantaneously. In the diffusion of a good solvent into a polymer, the properties of the polymer surface containing the penetrant molecules are different from those of the bulk polymer. For instance, even if the bulk of the polymer is glassy at *t* = 0, the surface might still exist in a rubbery state because of the differences in their levels of hydration. As the penetrant molecules diffuse from the rubbery outer surface to the glassy interior, a new equilibrium for the polymer chains is reached instantaneously as long as the polymer-solvent mixture at the advancing solvent front exists at equilibrium [24].

Figures 3 and 4 show that in the first hour, there is a gradual rise in OD of the gel while simultaneously its ID drops. While the mean annular thickness doubled in 48 h, it increased by up to 60% in just the first 15 min of immersion. We also note that the OD and ID growth curves in the first hour were roughly symmetrical, but were nearly identical after that. It was hypothesized based on symmetry and later proved by geometry (to within ±0.2 mm of accuracy) that in the first hour, the swelling can be visualized as equal and bidirectional (in both outward and inward directions) about a hypothetical concentric cylinder with radius (*b* − *a*)/2. The physical significance of this is that there are two advancing waterfronts at surfaces *r* = *a* and *r* = *b*, in the time interval of 0 < *t* < 1 h. This means that there must exist a diminishing region of glassy polymer that is sandwiched between 2 advancing ‘rubbery’ polymer-solvent boundaries. At *t* = 1 h, these two waterfronts meet at the center of the cylinder i.e. at *r* = (*b* − *a*)/2, so that the entire polymer bulk now exists in a more rubbery, hydrated state. After 1 hour, it was seen that both OD and ID increased steadily with time. This indicates that at times *t* > 1 h, the swelling is unidirectionally outwards. It can also be inferred that the rate of OD growth is slightly greater than the rate of ID growth after *t* = 1 h, as the wall thickness continues to increase slightly (~16%) over the next 47 h. The Peppas equation (Eq.(1) was used to calculate the values of transport exponent, *n*, and rate constant, *k*, for the first hour of swelling to characterize the nature of diffusion kinetics (Table 2).

**Table 2:** Diffusion parameters for a polymer sample of hollow cylindrical geometry, fully immersed in water for 1 hour (For the measurement of *n*, K in the following networks: *p* < 0.0001; calculated using the repeated measure ANOVA)

Polymer	*n*; 0 < *t* ≦ 3600 s	*K* (s^−*n*^)	Mode of diffusion	Diffusion constants
PAM 10%	0.79 ± 0.028	0.00038 ± 0.0015	Anomalous	–
PAM 20%	0.62 ± 0.005	0.00155 ± 8.19E-05	Anomalous	–
PAM 30%	0.64 ± 0.006	0.0035 ± 0.00075	Anomalous	–
PAM 40%	0.55 ± 0.0047	0.0021 ± 8.7E-06	~Fickian	*D* = (2.27 ± 0.1) × 10^−8^ cm^2^/s
PAM-PEGDA 20%				
(2% PEGDA)	0.93 ± 0.035	0.00011 ± 1.19E-05	Case II	*K*_o_ = (1.24 ± 0.032) × 10^−5^ cm/s; *t* = 80 min

As discussed in the previous section, a more concentrated gel has a reduced rate of water uptake. This indicates that *t*_d_ increases or the rate of diffusion decreases at higher polymer concentrations. Previous work done by S. R. Raghavan et. al. also shows that, for tubular hydrogels with identical composition, this characteristic time for diffusion depends strongly on the wall thickness as well [25]. Since the crosslinker ratio X is maintained as a constant, it can also be argued that *t*_r_ increases (rate of polymer chain relaxation decreases) only slightly with concentration, that too as a result of any physical bonding only such as chain entanglements, hydrogen bonding, etc. This theoretical behavior can be validated by the observed decreasing trend in *n* values with polymer concentration, in the first hour of swelling. Since n lies between 0.45 and 0.92 for all the PAM homopolymers, the diffusion model is characterized as anomalous, which suggests that *t*_r_–*t*_d_. The lower *n* values, however, imply that the rate-determining step in the anomalous diffusion processes in the homopolymers slowly shifts from being the rate of chain relaxation (for 10% PAM) toward the rate of diffusion (for 40% PAM) i.e. the initial (*t* < 1 h) mode of diffusion becomes increasingly Fickian, as the polymer concentration is increased. The PEGDA network being more hydrophilic exhibits the highest swelling ratio up to 2.5 h. The value of the transport exponent, *n* = 0.93, suggests it is purely Case II transport, justifying this high initial rate of water uptake. This also justifies the greater rate of growth in ID in the PEGDA network, compared to the 20% PAM homopolymer network. Another notable implication is that any minor increase in *v*_2_^0^ resulting from an increase in PEGDA content could circumvent the lower diffusion rates, at short times at least. This means that adding PEG chains into hydrogels could overcome the tradeoff between high rates of diffusion, and high linear swelling ratios at equilibrium. Around *t* = 1 h, the two advancing waterfronts must meet at *r* = (*b* − *a*)/2. This is validated by the fact that the average fractional water uptake in the PEGDA gels at this point is observed to be 20% (*M*_t_/*M*_∞_ = 0.2), in accordance with the rules for Case II transport i.e. the linear time relationship of water uptake is valid only for the first 15–20% of the net sorption process. Beyond this time scale it is expected that sorption shifts from being Case II dominant (*t*_r_ slightly greater than *t*_d_) to Case I or Fickian dominant (*t*_r_ < *t*_d_) for each network because of the plasticizing effect of water entering the network, resulting in higher rates of chain relaxation in response to the osmotic pressure.

### Determination of the diffusion parameters

Crank derived the general solutions to the differential equations governing diffusion through planar, spherical, and cylindrical geometries for some general boundary conditions [26]. In this study, polymers with hollow cylindrical/tube-like geometries were immersed in water completely to study their water transport properties. We assumed only radial diffusion and neglected any water flux in the thin *z*-direction. The boundary conditions were defined such that, for a given cylinder of internal radius ‘*a*’ and external radius ‘*b*’, both the inner (*r* = *a*) and outer surfaces (*r* = *b*) are maintained at the same constant concentration throughout. It must be noted that in our experiments, the solvent molecules not only penetrated the polymer but also swelled it. Thus, the boundary between the polymer and solvent varied with time, a factor not considered in Crank’s model. We used Peppas’ equations for a moving coordinate system to overcome this difficulty. It was noted by Lü and Bülow in 2000, that the uptake curves for samples with hollow cylindrical, and hollow planar geometries were nearly identical when the inner and outer surfaces were both permeable to diffusion [27]. They also showed that the equivalent radius/thickness, *R*, for a solid cylinder/plate to have the same diffusion behavior as a hollow cylinder/plate of set dimensions, OD = 2*b*, ID = 2*a*, is given by

3 $$R = \frac{{b - a}}{2}$$

Thus, for our hollow cylinder case, the fractional uptake function can be estimated by substituting the value of R into l, in the function for a solid plane. For 60% of total water uptake in Case I diffusion, this is given by—

4 $$\frac{{M_t}}{{M_\infty }} = 4\left( {\frac{{Dt}}{{l^2}}} \right)^{\frac{1}{2}}\left( {\frac{1}{{\pi ^{\frac{1}{2}}}} + 2\mathop {\sum}\limits_n^\infty {\left( { - 1} \right)^n} ierfc\frac{{nl}}{{2\sqrt {Dt} }}} \right)$$

where *M*_t_ is the mass of water absorbed in time *t*, *M*_∞_ is the mass of water absorbed at equilibrium, *D* is the diffusion coefficient and *l* is the thickness of the plane [18]. At short times, the relaxation term can be neglected and the Fickian contribution can be characterized by a t^0.5^ time dependence, as below—

5 $$\frac{{M_t}}{{M_\infty }} = 4\left( {\frac{{Dt}}{{\pi l^2}}} \right)^{\frac{1}{2}}$$

Similarly, for Case II diffusion, the fractional uptake function for a solid plane is given by -

6 $$\frac{{M_t}}{{M_\infty }} = \frac{{2k_0}}{{C_0l}}t$$

where *k*_0_ is the Case II relaxation constant, *C*_0_ is the equilibrium water concentration and l is the thickness of the plane. This equation is valid only till *t* = C_0_l / 2k_0_ which corresponds to the time taken for 15–20% of total water uptake [18]. We calculated the values of *k*_0_ and D from the rate constants calculated in Table 2, for the first hour of sorption, for the 40% PAM homopolymer and the PEGDA network using Eqs. 5 and 6, respectively. We note that these values are valid for approximately the first hour only. At greater timescales, the relaxation terms associated with these equations cause a significant deviation from purely Fickian or Case II behavior, and an asymptotic lowering in the values of *D* and *k*_0_ is expected. The diffusion behavior of the other networks is more complex to model quantitatively because the dynamics of anomalous diffusion in glassy polymers is still an ongoing research theme in the academic community.

#### Limits assessment

The hydrogels reported here exhibit desirable swelling characteristics in water and PBS, however, their performance in blood has not been evaluated in this work. However, the mode of fouling in clinically used biomaterials, and the strategies for designing non-fouling surfaces, has been studied extensively in the past [28]. Furthermore, the swelling tests here were performed at 25 °C (RT) as opposed to 37 °C. We’d expect that higher temperature evaluations would confirm other reported work that the equilibrium swelling ratio is directly proportional to the temperature of the solvent bath [29]. It has also been shown that the diffusion coefficient rises with temperature as well [30].

## Conclusions

An engineered proof of concept for the applicability of PAM-PEGDA network hydrogels as swellable catheters as an alternative to Aquavene® materials. We characterized the mass and dimensional swelling response for each of the cross-linked homo- and heterogeneous networks, and found a >100% increase in each dimension of the tubular gels (ID, OD, and H) at equilibrium, relative to their dry states. This is comparable to Aquavene® as early in vitro studies showed that a 20-gauge IV expanded to an 18-gauge IV and the flow rate increased 100% over the first hour of simulated insertion. This extent of linear swelling could correspond to a 4× increase in volumetric flow rate in vivo with the same gravity-based flow. The rate of change in ID lagged the rate of change in OD in all the gels tested. It was hypothesized that this lag could be circumvented by making either the outer or inner surfaces impermeable to solvent. By the superposition of the dynamic dimensional plots for the PAM homopolymer and PEGDA/AAM networks, the extent of linear swelling at equilibrium only depended on polymer volume fraction. Finally, it was found that the transport mechanism of water through the PAM homopolymers varies from Anomalous to Fickian diffusion as the concentration of AAm monomer is increased from 10–40% w/w. The diffusion coefficient of the 40% PAM network was calculated for the first hour of sorption, using the short-time Fickian approximation to be (2.27 ± 0.1) × 10^*−*^^8^ cm^2^/s. The diffusion coefficients for PAM homopolymers, containing 10–30% w/w monomer content, were harder to quantify because of the complex dynamics of anomalous diffusion in glassy polymers. Incorporating PEGDA into the PAM network raised the initial rate of water uptake drastically, as the water uptake mechanism changed to Case II diffusion. This change in transport mechanism was attributed to the high hydrophilicity of PEGDA, acting as both a comonomer and a macromolecular crosslinker. The rate of ID expansion and its associated lag time are related to the hydrophilicity of the polymer matrix and kinetics of diffusion. The Case II diffusion parameter, *k*_0_, was found to be (1.24 ± 0.032) × 10^−5^ cm/s. The linear relationship between time and water uptake associated with Case II diffusion was found to be valid up to *t* = 80 min. At longer times, sorption kinetics shifts toward a Fickian mode as the resins saturate, and the rate of chain relaxation increases from water plasticizing the gel. Thus, PEGDA cross-linked hydrogels seem applicable as swellable catheter alternatives to Aquavene® because of their high rates of swelling, linear expansion at short times such as those required for surgery, and widely reported biocompatibility. However, as the exact causes of Aquavene® hypersensitivity reactions remain unknown, more work needs to be conducted on these systems to investigate their bioactivity and mechanical viability.

## Supplementary information

Supplementary Information

## Abbreviation

IV: intravenous
w.r.t: with respect to
PAM: poly-Acrylamide
PEGDA: Poly-(Ethylene Glycol)- Diacrylate
PU: polyurethane
ID: inner diameter
OD: outer diameter
H: height
X: crosslinker mole-ratio
ANOVA: analysis of variance
RT: room Temperature
DI: deionized
IPA: isopropyl alcohol
MW: molecular weight
M(t): mass at time *t*
M(∞): mass at saturation
*k*: kinetic time constant of sorption
*n*: kinetic time exponent of sorption
v_2_^0^: polymer volume in the prepared state
*Q*_m_: mass swelling ratio
Q_f_: dilution degree after gel preparation
*ρ*: density of polymer
*a*: internal radius
*b*: external radius
*t*_d_: time of diffusion
*t*_r_: time for chain relaxation
*r*: (*b* − *a*)/2
*D*: diffusion coefficient of water into the hydrogel
ko: case II diffusion coefficient
